# Supplementary material for: Virtual staining from bright-field microscopy for label-free quantitative analysis of plant cell structures
Source: Plant Mol Biol. 2025 Jan 31;115(1):29. doi: 10.1007/s11103-025-01558-w (PMC11782351; doi:10.1007/s11103-025-01558-w)
Supplement: Supplementary file 1 — Supplementary file1 (DOCX 262 KB) [file 11103_2025_1558_MOESM1_ESM.docx]

Supplementary Figure


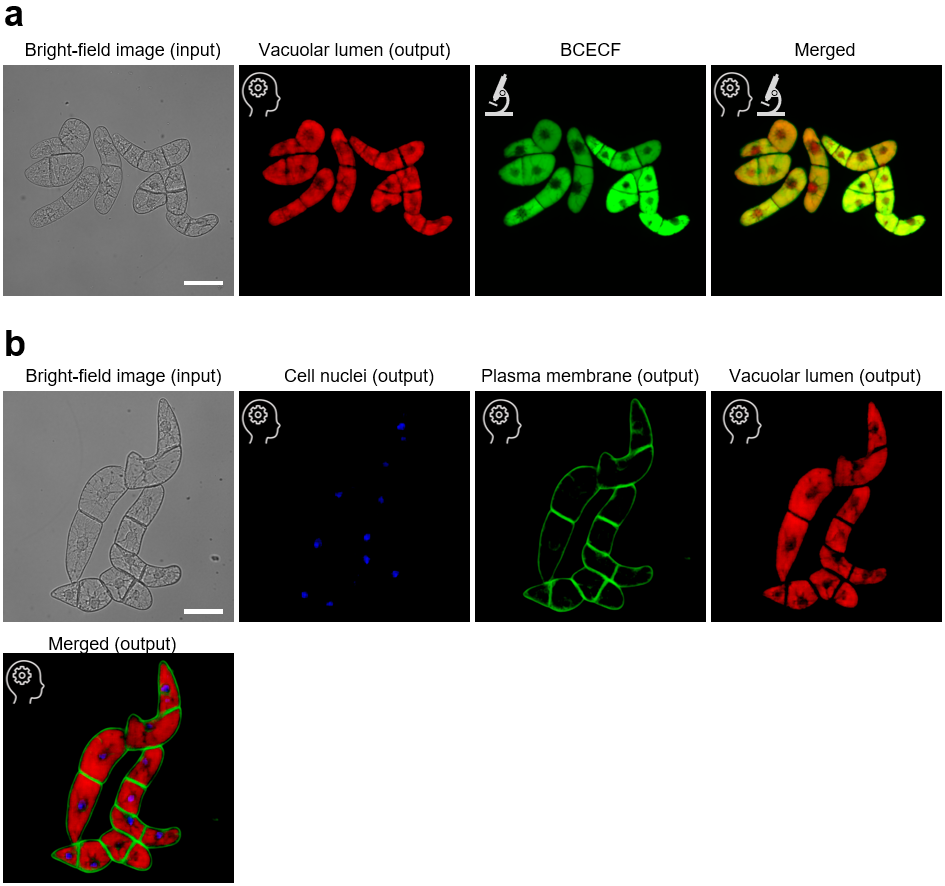


**Supplementary Fig. S1** Virtual staining of vacuoles in tobacco BY-2 cells. (a) Evaluation of virtual staining. Input bright-field image (far left) is followed by virtual staining output of the vacuolar lumen (second from left, red), confocal image of the vacuolar lumen labeled with the fluorescent dye BCECF (second from right, green), and merged image of virtual staining and fluorescence staining (far right). Virtually stained images closely align with corresponding confocal images. (b) Triple virtual staining of BY-2 cells. Bright-field image (far left) is followed by virtual staining outputs for cell nuclei (blue), plasma membrane (green), and vacuolar lumen (red). Lower panel displays a merged image of these three virtual stains. AI icon indicates virtual staining images, microscope icon denotes actual confocal microscopy images. Scale bars = 100 μm.
